# Analysis of the variable factors affecting changes in the blood concentration of cyclosporine before and after transfusion of red blood cell concentrate

**DOI:** 10.1186/s40780-021-00235-6

**Published:** 2022-02-01

**Authors:** Masashi Uchida, Natsumi Hanada, Shingo Yamazaki, Hirokazu Takatsuka, Chiaki Imai, Akari Utsumi, Yuki Shiko, Yohei Kawasaki, Takaaki Suzuki, Itsuko Ishii

**Affiliations:** 1 Division of Pharmacy, Chiba University Hospital, 1-8-1 Inohana, Chuo-ku, Chiba, 260-8677 Japan; 2 Graduate School of Pharmaceutical Sciences, Chiba University, 1-8-1 Inohana, Chuo-ku, Chiba, 260-8675 Japan; 3 Biostatics Section, Clinical Research Center, Chiba University Hospital, 1-8-1 Inohana, Chuo-ku, Chiba, 260-8677 Japan

**Keywords:** Cyclosporine, Red blood cell transfusion, Therapeutic drug monitoring, Hematopoietic stem cell transplantation

## Abstract

**Background:**

The blood concentration of cyclosporine (CyA) is frequently elevated following the transfusion of red blood cell concentrate (RCC) to patients after allogeneic hematopoietic stem cell transplantation (HSCT). The aim of this retrospective study was to identify the variable factors affecting changes in the blood concentration of CyA before and after transfusion of RCC.

**Methods:**

We enrolled 105 patients (age, 5–66 years) who received both CyA and transfusion after HSCT. The ratio of the measurement after transfusion to the measurement before transfusion was calculated for the hematocrit and blood concentration/dose ratio of CyA (termed the HCT ratio and the CyA ratio, respectively).

**Results:**

The blood concentration/dose ratio of CyA was increased after transfusion compared with before transfusion (*P* < 0.001). The HCT ratio was significantly correlated with the CyA ratio (*P* = 0.23, *P* < 0.001). The HCT ratio, concomitant medication that could elevate CyA concentration after RCC transfusion, and difference in the alkaline phosphatase level between before and after transfusion (ΔALP) were explanatory variables associated with the variation in the CyA ratio. There was no correlation between the CyA concentration after transfusion and the change in the estimated glomerular filtration rate.

**Conclusions:**

A change in the blood concentration/dose ratio of CyA was found to be associated with a change in the HCT, concomitant medication that could elevate CyA concentration after RCC transfusion, and ALP levels. If the HCT level rises significantly after RCC transfusion, clinicians and pharmacists should pay attention to changes in the blood CyA concentration.

**Supplementary Information:**

The online version contains supplementary material available at 10.1186/s40780-021-00235-6.

## Background

Cyclosporine (CyA) is an immunosuppressive drug used for prophylaxis of graft-versus-host-disease (GVHD) after allogeneic hematopoietic stem cell transplantation (HSCT) [1–3]. Appropriate CyA dosing is essential to improve its effectiveness and decrease associated adverse effects, including hypertension and nephrotoxicity [4–6]. Because CyA exhibits large pharmacokinetic variability, its blood concentrations in patients must be routinely monitored. The whole blood concentration of CyA is measured as part of therapeutic drug monitoring in daily practice because the blood-to-plasma ratio of CyA is affected by the temperature at which the whole blood sample is handled, as well as by hematocrit (HCT) and plasma lipid levels [7, 8].

Patients generally have a low HCT level after HSCT and receive transfusions of red blood cell concentrate (RCC). We frequently observe elevated blood CyA concentrations in patients after RCC transfusion. CyA binds to cyclophilin, which is expressed by T lymphocytes and erythrocytes [9, 10]. In human blood in vitro, 40 to 50% of CyA is distributed to erythrocytes [11]. 10 to 20% of CyA is found in leucocytes, and 30 to 40% is found in the plasma [11]. Most CyA in human plasma is associated with lipoproteins [8, 11]. Population pharmacokinetic (PPK) studies have demonstrated that the HCT level is inversely correlated with CyA clearance in HSCT patients [12–14] and in recipients of other types of transplants [15]. Although these reports suggested that RCC transfusion has some effects on the blood CyA concentration, the variable factors affecting changes in the blood concentration of CyA before and after transfusion of RCC have not been understood.

The aim of this retrospective study was to identify the variable factors affecting changes in the blood concentration of CyA before and after transfusion of RCC.

## Methods

### Patient selection and data collection

The present study enrolled inpatients at Chiba University Hospital who received both CyA (continuous intravenous infusion [Sandimmun® injection] or oral administration [Neoral® capsule or its generic brand]) and RCC transfusion after allogeneic HSCT between January 2012 and December 2018.

Data were retrospectively obtained from the electronic medical record system. The following information was obtained for each patient: age, sex, primary disease, donor type and stem cell, body weight measured closest to the day of the RCC transfusion, CyA dose, HCT levels, white blood cell (WBC) counts, aspartate aminotransferase (AST) levels, alanine aminotransferase (ALT) levels, alkaline phosphatase (ALP) levels, total bilirubin (Tbil) levels, serum albumin (Alb) levels, serum urea nitrogen (UN) levels, serum creatinine (Scr) levels, serum potassium (K) levels, RCC transfusion volumes, concomitant medications, and blood concentrations of CyA. The blood concentrations of CyA were measured using a chemiluminescent immunoassay (Architect® system; Abbott, Tokyo, Japan). When the CyA concentration was measured on the same day as the transfusion, the electronic medical records were checked to confirm that the blood for CyA measurement was collected before the transfusion.

### Data analysis

In this study, administration of CyA was initiated after HSCT with continuous intravenous infusion, intravenous infusion for 10 h a day, or intravenous infusion for 3 h twice a day. The blood concentration of CyA was measured 1 to 3 times a week and the CyA dose was changed if necessary. The blood for measurement of CyA concentration was collected around 7 am, and collected before administration except for continuous intravenous infusion. The cases for analysis were defined as follows: cases in which the blood CyA concentration was measured within 7 days before and after a single RCC transfusion, and cases in which the blood CyA concentration was measured twice within 7 days and two or more RCC transfusions were performed during the same period (Fig. S1). The elimination half-life of CyA shows relatively high interpatient variability [16]: the half-life has been reported as 6.8 ± 2.5 h (intravenous administration) and 11.3 ± 6.8 h (oral administration) [17], 15.8 ± 8.4 h (intravenous administration) [18], and 5.6 to 34.6 h (oral and intravenous administration) [19]. Therefore, we considered the half-life of CyA to be about 12 h and that it would take 3 days for the blood CyA concentration to reach a steady state for the first time. Blood concentrations of CyA were excluded from the analysis if they were measured within 3 days after the start of administration. The cases in which the route of administration was changed from intravenous infusion to oral administration before and after RCC transfusion were also excluded from the analysis.

For this study, two blood concentration/dose (C/D) ratios for CyA were calculated, in which the dose was adjusted using the body weight [i.e., (ng/mL)/(mg/kg)/day]: the blood concentration/dose before transfusion (Cb/Db) and the blood concentration/dose after transfusion (Ca/Da). (Ca/Da) was then divided by (Cb/Db) to obtain what we termed the CyA ratio. When the CyA dose was changed before or after the RCC transfusion, the CyA ratio was corrected as follows: once the blood concentration of CyA reached a steady state, it was expected to require less than 3 days to reach a steady state again after the dose change according to the pharmacokinetics. We assumed that the blood CyA concentration would be stable 2 days after a dose change. If the interval between the dose change and the concentration measurement was 2 days or more, the new CyA dose was used in our calculations.

The estimated glomerular filtration rate (eGFR) of patients older than 18 years of age was calculated using the formula for the Japanese population [20]. The clinical laboratory data used for the analysis were obtained on the same day as the CyA concentrations. Differences (for eGFR, and AST, ALT, ALP, Tbil, Alb, UN, and K levels) and ratios (for the HCT level and WBC count) between before and after transfusion were calculated for each case.

### Statistical analysis

The blood CyA concentration, C/D ratio of CyA, and clinical laboratory data before and after transfusion were compared using the Wilcoxon signed-rank test.

Correlations between the CyA ratio and various factors, except sex and concomitant medications, were measured using Spearman’s correlation coefficient test. The correlation ratio (η) was calculated for sex and concomitant medications. Then, multiple regression analysis was performed with both forward selection and backward selection methods. For HCT levels and WBC counts which related to the distribution of CyA in the blood, HCT ratio and WBC ratio were used as independent variables. For clinical laboratory data related to liver function which could be responsible for CyA metabolism and excretion, the differences before and after transfusion were selected as independent variables. Sex, age, body weight, units of RCC transfusion, and concomitant medications were also used as independent variables and the CyA ratio was used as the response variable. For sex and concomitant medications, male and no concomitant medication affecting CyA concentration after RCC transfusion were served as the reference group. SPSS version 24 (IBM Corp., Armonk, NY) was used for the analyses. Statistical significance was defined as *P* < 0.05.

## Results

### Characteristics of the analyzed cases

The characteristics of 105 patients (age, 5–66 years) are summarized in Table 1. A total of 580 cases were analyzed. The median number of cases per patient was 4 (range, 1–38). The median interval between measurements of the CyA concentration before and after transfusion was 3 (range, 1–7) days. The most frequent RCC transfusion volume was 2 units (504 cases, 86.9%). RCC transfusion was performed within 4 weeks after HSCT in 367 cases (63.3%), and the median interval was 21 days (range, 2–218 days). CyA was intravenously administered in 551 cases (i.e., continuous intravenous infusion in 404 cases, intravenous infusion for 10 h a day in 138 cases, and intravenous infusion for 3 h twice a day in 9 cases) and orally in 29 cases. The CyA dose was unaltered between the measurements of the CyA concentration before and after transfusion in 252 cases.

**Table 1:** Patient characteristics

Characteristic	
	Median (range)
Age (years)	49 (5–66)
Sex	no. (%)
Male	60 (57.1)
Female	45 (42.9)
	Median (range)
Body weight (kg)	61.2 (19.2–89.0)
Primary disease	no. (%)
Acute leukemia	57 (54.3)
Myelodysplastic syndrome	22 (21.0)
Malignant lymphoma	15 (14.3)
Chronic leukemia	5 (4.8)
Aplastic anemia	2 (1.9)
Chronic active Epstein–Barr virus infection	2 (1.9)
Myeloid sarcoma	1 (1.0)
Secondary myelofibrosis	1 (1.0)
Donor type and stem cell	no. (%)
Related, bone marrow	16 (13.8)
Related, peripheral blood	19 (16.4)
Unrelated, bone marrow	31 (26.7)
Unrelated, cord blood	44 (37.9)
Unrelated, peripheral blood	6 (5.2)
Units of RCC transfusion (unit)	no. (%)
2	504 (86.9)
4	55 (9.5)
6	11 (1.9)
8	4 (0.7)
10	1 (0.2)
12	4 (0.7)
14	1 (0.2)

### Relationship between various factors and the C/D ratio of CyA before and after transfusion

**Fig. 1:**
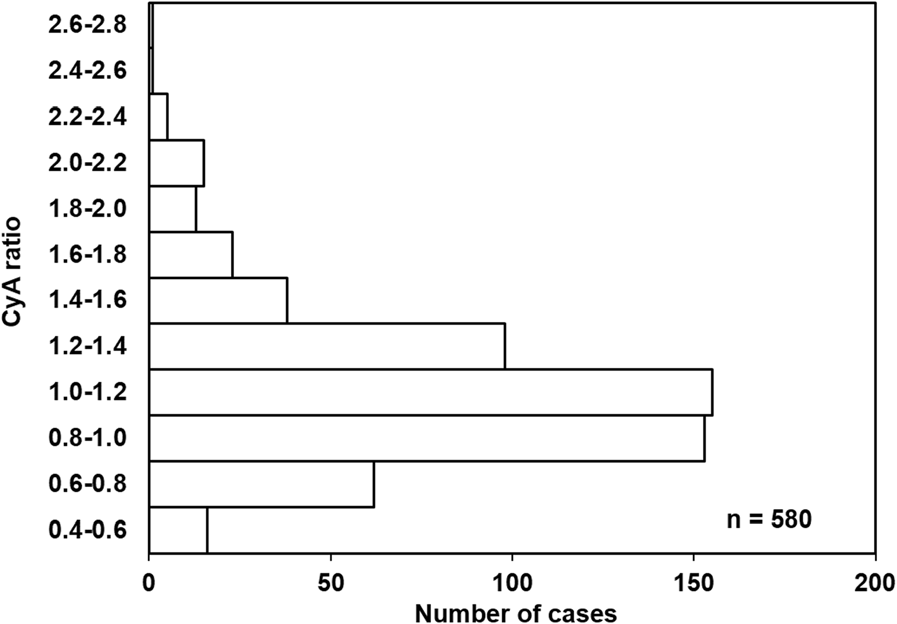
Histogram of the CyA ratio

The HCT level and C/D ratio of CyA were significantly increased after RCC transfusion (Table S1). The median CyA ratio was 1.08 (range, 0.43–2.64), and 406 cases (70.0%) had a ratio within the range of 0.8 to 1.4 (Fig. 1). Correlations between the CyA ratio and various patient characteristics or clinical laboratory data were examined (Table 2). The CyA ratio showed significant positive correlations with the HCT level after transfusion and with the HCT ratio (see also Fig. S2). The CyA ratio was also correlated with WBC, AST, ALT, ALP, Tbil, and Alb values. In 39 cases, medication that could affect the blood CyA concentration (azole antifungal agent, amlodipine, nifedipine, metronidazole, or deferasirox) was initiated or discontinued between the CyA concentration measurements before and after transfusion. When all cases were analyzed, these concomitant medications were correlated with the variation in the CyA ratio (Table 2).

**Table 2:** Relationship between the CyA ratio and various factors

	All cases			Cases in which the CyA dose was not changed between before and after RCC transfusion		
	no.	*ρ* or η	*P*	no.	*ρ* or η	*P*
Sex^a^	580	0.011	0.784	252	0.053	0.402
Age	580	−0.057	0.173	252	−0.027	0.672
Body weight	563	−0.022	0.597	242	0.000	0.997
Units of RCC transfusion	580	0.026	0.534	252	0.056	0.372
HCTb	574	−0.056	0.184	251	−0.026	0.681
HCTa	571	0.187	< 0.001^b^	245	0.240	< 0.001^b^
HCT ratio	565	0.234	< 0.001^b^	244	0.273	< 0.001^b^
WBCb	475	0.123	< 0.01^b^	215	0.110	0.108
WBCa	500	0.091	0.041^b^	214	0.105	0.125
WBC ratio	447	−0.002	0.970	198	0.007	0.917
ASTb	569	0.073	0.084	247	0.129	0.042^b^
ASTa	567	0.120	< 0.01^b^	242	0.161	0.012^b^
ΔAST	558	0.064	0.128	239	0.144	0.026^b^
ALTb	569	0.049	0.245	247	0.081	0.202
ALTa	567	0.080	0.057	242	0.142	0.027^b^
ΔALT	558	0.095	0.025^b^	239	0.176	< 0.01^b^
ALPb	539	0.048	0.268	232	0.090	0.174
ALPa	529	0.064	0.141	224	0.118	0.079
ΔALP	508	0.102	0.021^b^	216	0.146	0.032^b^
Tbil b	561	−0.050	0.237	246	−0.027	0.675
Tbil a	563	−0.041	0.330	242	0.043	0.507
ΔTbil	548	0.048	0.259	238	0.135	0.037^b^
Alb b	443	−0.055	0.250	184	−0.143	0.052
Alb a	447	0.058	0.224	182	0.036	0.626
ΔAlb	356	0.228	< 0.001^b^	139	0.376	< 0.001^b^
Concomitant medication^a^:						
No concomitant medication affecting Ca	541	0.134	0.015^b^	241	0.059	0.834
Concomitant medication that could elevate Ca^c^	25			5		
Concomitant medication that could lower Ca^d^	8			5		
Concomitant medication that could elevate or lower Ca^e^	6			1		

*RCC* red blood cell concentrate, *HCT* hematocrit, *WBC* white blood cell count, *AST* aspartate aminotransferase, *ALT* alanine aminotransferase, *ALP* alkaline phosphatase, *Tbil* total bilirubin, *Alb* serum albumin, *CyA* cyclosporine, *b* before RCC transfusion, *a* after RCC transfusion, *Ca* blood concentration of cyclosporine after RCC transfusion. ^a^Correlation ratio (η) was calculated. ^b^*P* < 0.05; ^c^Initiation of azole antifungal agent, amlodipine, nifedipine, or metronidazole. ^d^Initiation of deferasirox, discontinuation of voriconazole, amlodipine or metronidazole, or switch from voriconazole to fluconazole. ^e^Switch from itraconazole to voriconazole, switch from amlodipine to nifedipine, initiation of itraconazole and deferasirox, administration or discontinuation of azole antifungal agent only for 1 or 2 days

In a multiple regression analysis, we used sex, age, body weight, units of RCC transfusion, HCT ratio, WBC ratio, ΔAST, ΔALP, ΔTbil, and concomitant medications as independent variables. ΔALT was excluded as there was a strong correlation between ΔAST and ΔALT (i.e., *ρ* = 0.72; *P* < 0.001 for all cases, and *P* = 0.64; *P* < 0.001 for cases in which the CyA dose was not changed between before and after RCC transfusion, respectively). ΔAlb was excluded because of the relatively small number of cases. Multiple regression analysis revealed that the HCT ratio and concomitant medication that could elevate CyA concentration after RCC transfusion were explanatory variables associated with the variation in the CyA ratio (Table 3). In addition, ΔALP was explanatory variable for cases in which the CyA dose was not changed between before and after RCC transfusion (Table 3).

**Table 3:** Multiple regression analysis to identify factors associated with the variation in the CyA ratio

	Variable	no.	Partial regression coefficient	95% CI	Standardized partial regression coefficient	*P*
All cases	HCT ratio	381	0.389	0.153–0.624	0.162	< 0.01
	Concomitant medication that could elevate Ca^a^	381	0.279	0.129–0.428	0.183	< 0.001
Cases in which the CyA dose was not changed between before and after RCC transfusion	HCT ratio	165	0.479	0.166–0.792	0.227	< 0.01
	ΔALP	165	0.001	0.0001–0.001	0.168	0.027
	Concomitant medication that could elevate Ca^a^	165	0.321	0.001–0.640	0.147	0.049

*CI* confidence interval, *CyA* cyclosporine, *HCT* hematocrit, *Ca* blood concentration of cyclosporine after RCC transfusion, *ALP* alkaline phosphatase. ^a^ No concomitant medication affecting Ca in Table 2 served as the reference group

### Relationship between blood concentration of CyA and renal function after transfusion

**Fig. 2:**
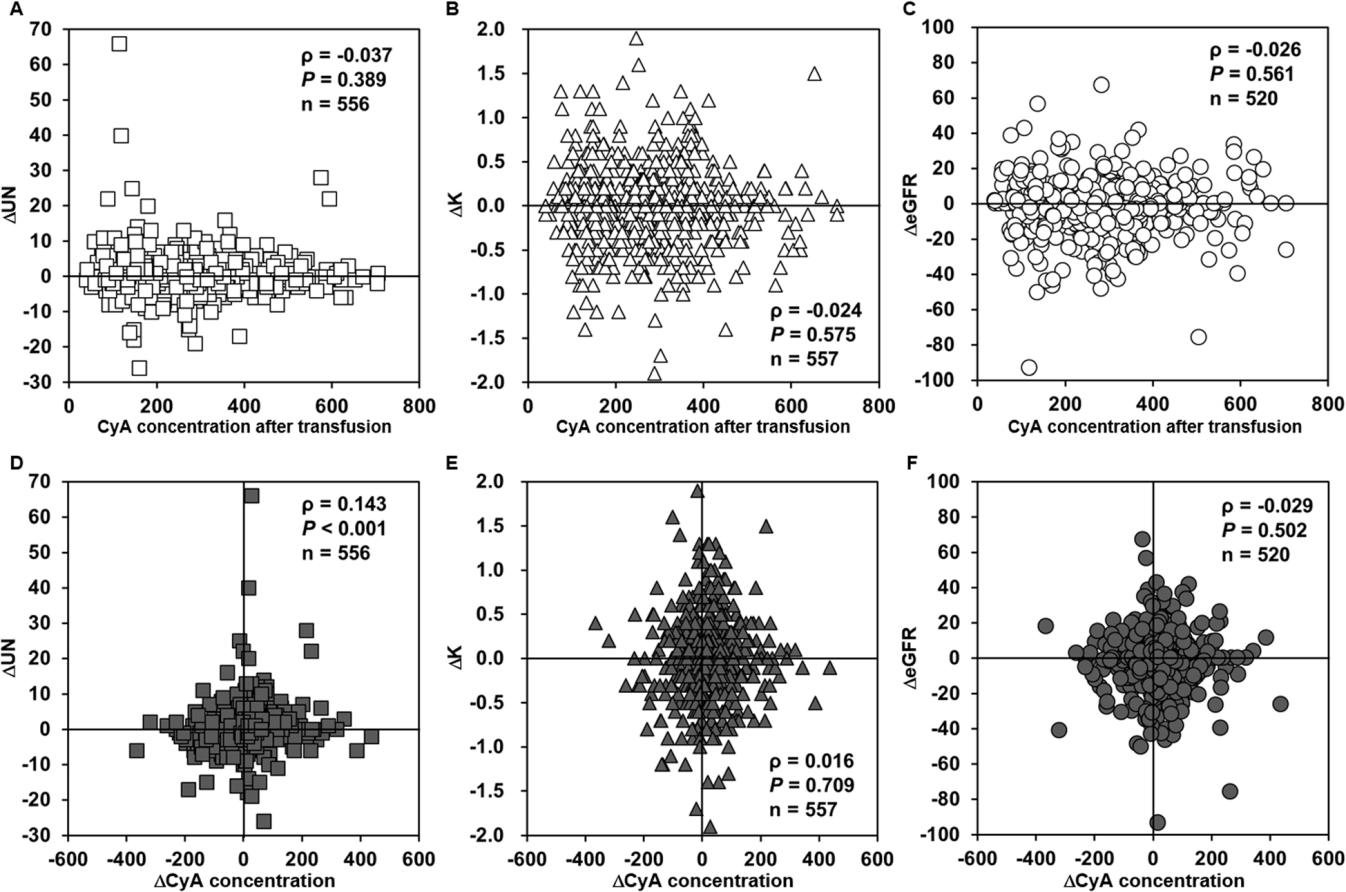
Relationship between blood concentration of CyA and renal function after transfusion. Relationship between CyA concentration after transfusion and ΔUN (A), ΔK (B), and ΔeGFR (C). Relationship between ΔCyA concentration and ΔUN (D), ΔK (E), and ΔeGFR (F). CyA, cyclosporine; UN, urea nitrogen; K, serum potassium; eGFR, estimated glomerular filtration rate

There was a significant difference in UN and eGFR levels before and after transfusion for all cases (Table S1). Relationship between blood concentration of CyA after transfusion and renal function or related laboratory data were examined. No correlations were observed between the blood concentration of CyA after transfusion and the ΔUN, ΔΚ and ΔeGFR (Fig. 2A-C). There was a weak positive correlation between the difference in the CyA concentration before and after transfusion (ΔCyA concentration) and the ΔUN (Fig. 2D). ΔCyA concentration was not correlated with the ΔK or ΔeGFR (Fig. 2E and F).

## Discussion

This study identified a significant increase in the C/D ratio of CyA between before and after RCC transfusion and found that the HCT ratio, concomitant medication that could elevate CyA concentration after RCC transfusion, and ΔALP were associated with the variation in the CyA ratio.

Both in vitro and clinical studies have well established that CyA is associated mainly with erythrocytes in blood and that HCT can alter the CyA clearance. In vitro, 40 to 50% of CyA is distributed to erythrocytes [11]. CyA binding to erythrocytes at a HCT of 50% is estimated to be saturated at a concentration of 3000 ng/mL, which is much higher than the therapeutic range [10]. From the pharmacokinetic point of view, based on in vitro experiments, an increase in HCT leads to an increase in the blood-to-plasma CyA concentration ratio, resulting in a decrease in the blood clearance of CyA [15, 21]. Some clinical studies in HSCT patients demonstrated that a high HCT level was significantly associated with low clearance of CyA [12–14]. These reports are consistent with our findings that the C/D ratio of CyA increased after RCC transfusion and that the HCT ratio was associated with the variation in the CyA ratio.

CyA is metabolized mainly in the liver by cytochrome P450 (CYP) 3A4 [22]. Various drugs induce or inhibit CYP3A and alter the clearance of CyA [22, 23]. PPK models in HSCT patients have revealed that azole antifungal agents affect the clearance of CyA [13, 14]. Consistent with these reports, concomitant medication that could elevate CyA concentration after RCC transfusion (i.e., initiation of azole antifungal agent, amlodipine, nifedipine, or metronidazole) were correlated with the variation in the CyA ratio in our multivariate analysis. This indicates that we should pay attention to changes in the blood CyA concentration when these drugs which could alter the clearance of CyA were initiated or discontinued. On the other hand, concomitant medication that could lower CyA concentration after RCC transfusion were not correlated with the variation in the CyA ratio. This may be partially because any medications that could change the blood CyA concentration had been discontinued or because the patients had been switched to other drugs prior to the HSCT, and there were only a small number of cases in which these drugs were initiated or discontinued between before and after transfusion.

Most metabolites of CyA are excreted in the bile [22]. CyA clearance has been reported to be lower in HSCT patients with an elevated serum bilirubin level than in those with a normal level, suggesting that biliary tract abnormalities could affect CyA clearance [24]. In the present study, the ΔALP was associated with the variation in the CyA ratio in cases in which the CyA dose was not changed between before and after RCC transfusion. ALP is an enzyme derived mainly from the liver and bones. A rise in ALP occurs with cholestasis, particularly obstructive jaundice, and with diseases of the skeletal system [25, 26]. The elevated ALP level may reflect the lower CyA elimination and higher CyA ratio in this study, although the ΔTbil was correlated with the CyA ratio only in univariate analysis for the cases in which the CyA dose was not changed between before and after transfusion. However, Eljebari et al. reported that ALP and bilirubin levels, as well as AST and ALT levels, were not covariates of CyA clearance in PPK analysis in HSCT patients [27]. In addition, the relationship between RCC transfusion and the ALP level has not been well understood, although it has been reported that acute hypophosphatasemia was observed after massive transfusions (> 20–100 units of blood product) [28]. In the present study, there was a slight increase in ALP level after transfusion (Table S1), and it could not be denied that the ALP level may have increased due to the adverse effect of increased CyA concentration after transfusion. Further studies will need to examine the relationship between CyA clearance and ALP.

In our previous study, there was a correlation between the change in the C/D ratio of tacrolimus (termed “TCR ratio”) and age or body surface area (BSA) [29]. We also found a correlation between the HCT ratio and age or BSA. Therefore, the results suggest that a smaller body size in children could result in a greater HCT ratio and thus a greater TCR ratio than in adults. However, age and BSA were not associated with the variation in the CyA ratio in the present study (data not shown for BSA). This might be because only 10 of the total 580 cases were pediatric cases (age < 15 years). The blood concentration of CyA may tend to increase in children or patients with a smaller BSA after RCC transfusion, similar to tacrolimus.

In the present study, the correlation coefficient between CyA ratio and various factors used for the analysis was small (*ρ* < 0.3), indicating that correlation was weak (Table 2). This suggests that these factors including HCT ratio could not fully explain the variation in the CyA ratio. Further studies will need to quantitatively predict the changes in the blood concentration of CyA before and after RCC transfusion.

Nephrotoxicity is one of the most important adverse effects of CyA. Some reports suggested the existence of a correlation between the blood or serum trough concentration of CyA and nephrotoxicity [30, 31], although other reports found no correlation between the two [32, 33]. The CyA trough concentration and K level have also been positively correlated [33]. In the present study, although there was a weak correlation between ΔCyA concentration and ΔUN, both CyA concentration after transfusion and ΔCyA concentration were not correlated with the ΔeGFR or ΔK (Fig. 2), suggesting that renal function is unlikely to be greatly influenced by RCC transfusion in the short term. It is also suggested that other pharmacokinetic parameters such as the area under the blood concentration time curve or the plasma or unbound concentrations of CyA are associated with the change in the renal function, although we have no data to estimate these parameters.

This study has the following limitations. First, factors such as concomitant medications may also affect the changes in renal function. Several drugs that could affect renal function were used in combination with CyA in patients receiving HSCT, and it was difficult to clarify the effects of concomitant medications on renal function. Second, we were unable to consider lipoproteins in this study. Most CyA in human plasma is associated with lipoproteins [8, 11], and triglycerides and plasma cholesterol levels have been reported to be significant covariates influencing CyA clearance [14, 34]. Lipoprotein contents may have affected the CyA ratio in this study. Third, we did not evaluate the long-term influence on stem cell engraftment, GVHD, and renal function.

## Conclusions

The present study identified a significant increase in the C/D ratio of CyA between before and after RCC transfusion. The HCT ratio, concomitant medication that could elevate CyA concentration after RCC transfusion, and ΔALP were associated with a variation in the CyA ratio. Renal function is unlikely to be greatly influenced by RCC transfusion. Clinicians and pharmacists should pay attention to changes in the blood CyA concentration if the HCT level rises significantly after RCC transfusion.

## Supplementary Information

**Additional file 1.** Fig. S1 Methods used in the analysis. The clinical laboratory data used for the analysis were obtained on the same day as the CyA concentrations. Differences and ratios between before and after transfusion were calculated for each case. Medications that could affect the blood concentration of CyA (azole antifungal agents, amlodipine, nifedipine, metronidazole, or deferasirox) that were initiated or discontinued during the period are indicated in gray in the time course and were analyzed as concomitant medications in Table 2. RCC, red blood cell concentrate; D, dose of CyA; C, blood concentration of CyA; HCT, hematocrit; WBC, white blood cell count; AST, aspartate aminotransferase; ALT, alanine aminotransferase; ALP, alkaline phosphatase; Tbil, total bilirubin; Alb, serum albumin; UN, urea nitrogen; eGFR, estimated glomerular filtration rate; K, serum potassium; CyA, cyclosporine; b, before RCC transfusion; a, after RCC transfusion.

**Additional file 2.** Table S1. Changes in the blood concentration of CyA and clinical laboratory data between before and after transfusion.

**Additional file 3.** Fig. S2 Relationship of the CyA ratio with the HCT ratio. A, All cases. B, Cases in which the CyA dose was not changed between before and after RCC transfusion. HCT, hematocrit; CyA, cyclosporine; RCC, red blood cell concentrate.

## Data Availability

The datasets used and/or analyzed during the current study are available from the corresponding author on reasonable request.
