# Supplementary material for: Analysis of the variable factors affecting changes in the blood concentration of cyclosporine before and after transfusion of red blood cell concentrate
Source: J Pharm Health Care Sci. 2022 Feb 1;8:4. doi: 10.1186/s40780-021-00235-6 (PMC8805225; doi:10.1186/s40780-021-00235-6)
Supplement: Supplementary file 1 — Additional file 1. Fig. S1 Methods used in the analysis. The clinical laboratory data used for the analysis were obtained on the same day as the CyA concentrations. Differences and ratios between before and after transfusion were calculated for each case. Medications that could affect the blood concentration of CyA (azole antifungal agents, amlodipine, nifedipine, metronidazole, or deferasirox) that were initiated or discontinued during the period are indicated in gray in the time course and were analyzed as concomitant medications in Table 2. RCC, red blood cell concentrate; D, dose of CyA; C, blood concentration of CyA; HCT, hematocrit; WBC, white blood cell count; AST, aspartate aminotransferase; ALT, alanine aminotransferase; ALP, alkaline phosphatase; Tbil, total bilirubin; Alb, serum albumin; UN, urea nitrogen; eGFR, estimated glomerular filtration rate; K, serum potassium; CyA, cyclosporine; b, before RCC transfusion; a, after RCC transfusion. [file 40780_2021_235_MOESM1_ESM.docx]

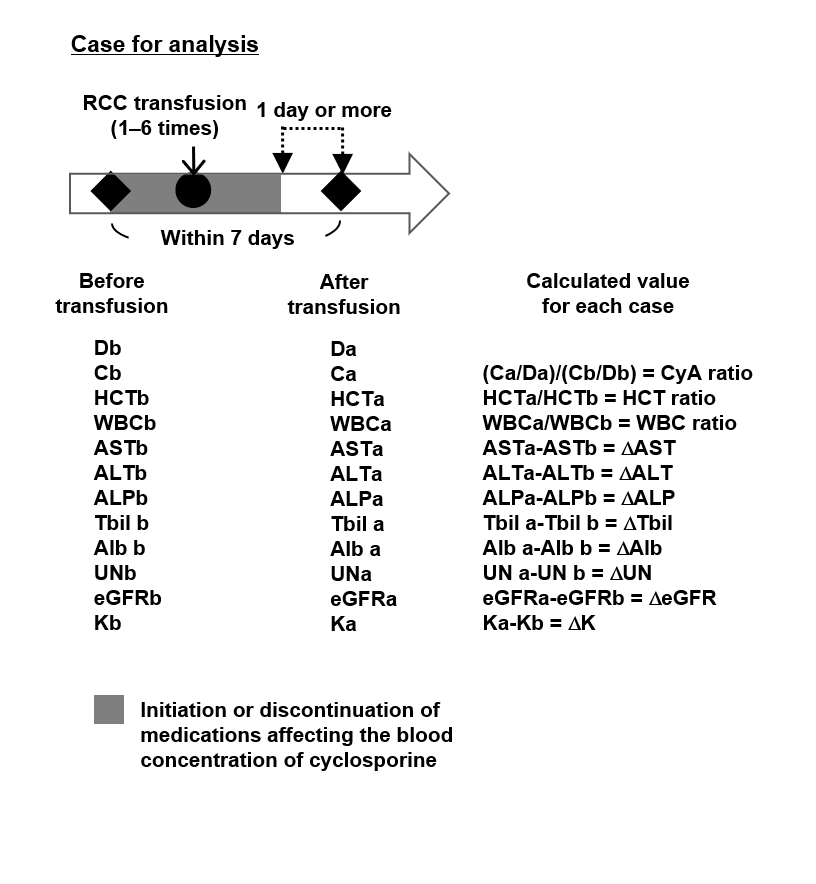


**Fig. S1 Methods used in the analysis.**

The clinical laboratory data used for the analysis were obtained on the same day as the CyA concentrations. Differences and ratios between before and after transfusion were calculated for each case. Medications that could affect the blood concentration of CyA (azole antifungal agents, amlodipine, nifedipine, metronidazole, or deferasirox) that were initiated or discontinued during the period are indicated in gray in the time course and were analyzed as concomitant medications in Table 2. RCC, red blood cell concentrate; D, dose of CyA; C, blood concentration of CyA; HCT, hematocrit; WBC, white blood cell count; AST, aspartate aminotransferase; ALT, alanine aminotransferase; ALP, alkaline phosphatase; Tbil, total bilirubin; Alb, serum albumin; UN, urea nitrogen; eGFR, estimated glomerular filtration rate; K, serum potassium; CyA, cyclosporine; b, before RCC transfusion; a, after RCC transfusion.
