# Supplementary material for: Analysis of the variable factors affecting changes in the blood concentration of cyclosporine before and after transfusion of red blood cell concentrate
Source: J Pharm Health Care Sci. 2022 Feb 1;8:4. doi: 10.1186/s40780-021-00235-6 (PMC8805225; doi:10.1186/s40780-021-00235-6)
Supplement: Supplementary file 2 — Additional file 2. Table S1. Changes in the blood concentration of CyA and clinical laboratory data between before and after transfusion. [file 40780_2021_235_MOESM2_ESM.docx]

**Table S1. Changes in the blood concentration of CyA and clinical laboratory data between before and after transfusion.**
