# Supplementary material for: Analysis of the variable factors affecting changes in the blood concentration of cyclosporine before and after transfusion of red blood cell concentrate
Source: J Pharm Health Care Sci. 2022 Feb 1;8:4. doi: 10.1186/s40780-021-00235-6 (PMC8805225; doi:10.1186/s40780-021-00235-6)
Supplement: Supplementary file 3 — Additional file 3. Fig. S2 Relationship of the CyA ratio with the HCT ratio. A, All cases. B, Cases in which the CyA dose was not changed between before and after RCC transfusion. HCT, hematocrit; CyA, cyclosporine; RCC, red blood cell concentrate. [file 40780_2021_235_MOESM3_ESM.docx]

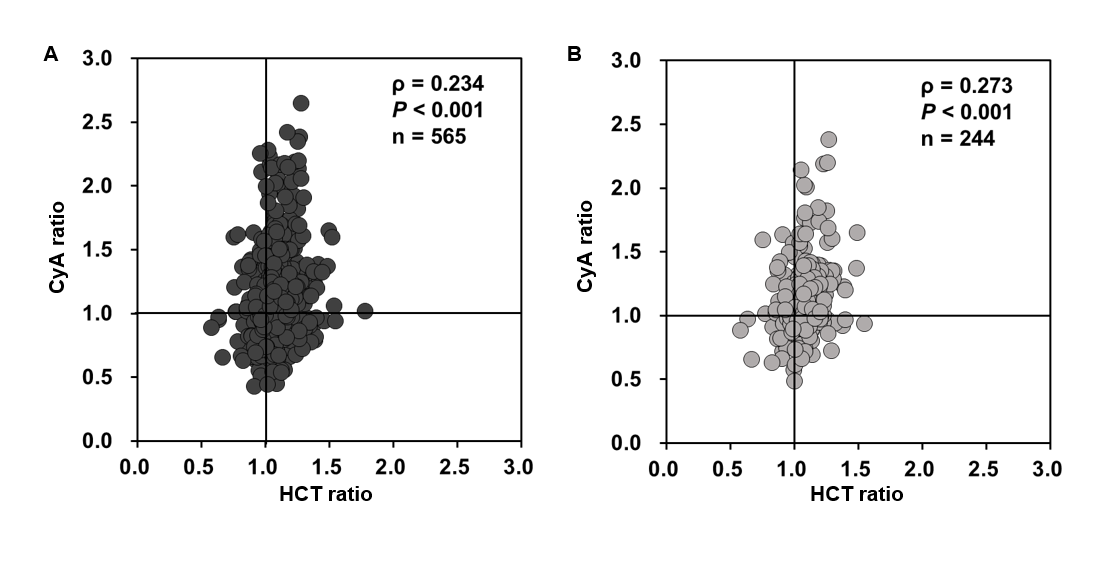


**Fig. S2 Relationship of the CyA ratio with the HCT ratio.**

A, All cases. B, Cases in which the CyA dose was not changed between before and after RCC transfusion. HCT, hematocrit; CyA, cyclosporine; RCC, red blood cell concentrate.
